# Feasibility and acceptability of integrating mass distribution of azithromycin to children 1–11 months into a trachoma mass drug administration campaign to reduce child mortality in Côte d’Ivoire

**DOI:** 10.1371/journal.pgph.0003426

**Published:** 2024-07-10

**Authors:** Lisa Dulli, Fatoumata Touré, Adam Djima Mama, Emily Evens, Kate Murray, Norbert N’goran Djè, Stéphane Koné, Pat Sadate-Ngatchou, Anoma Bovary, Marga Eichleay, Milenka Jean-Baptiste, Aboulaye Méité

**Affiliations:** 1 Global Health and Population Business Unit, FHI 360, Durham, North Carolina, United States of America; 2 Global Health and Population Business Unit, FHI 360, Abidjan, Côte d’Ivoire; 3 The Côte d’Ivoire National Neglected Tropical Diseases Program (Programme National de Lutte contre les Maladies Tropicales Négligées à Chimiothérapie Préventive), Abidjan, Côte d’Ivoire; 4 Department of Health Behavior, Gillings School of Global Public Health, University of North Carolina at Chapel Hill, Chapel Hill, North Carolina, United States of America; University of California San Francisco, UNITED STATES

## Abstract

Evidence suggests that bi-annual mass drug administration (MDA) of single-dose azithromycin to 1–11 month-old children reduces child mortality in high child-mortality settings. Several countries conduct annual MDAs to distribute azithromycin to individuals ages 6 months and older to prevent trachoma infection. This study examined the feasibility and acceptability of reaching 1–11 months-old children during a trachoma MDA in Côte d’Ivoire by extending azithromycin distribution to infants 1–5 months old during the campaign. In November 2020, the study piloted single-dose azithromycin for 1–5 month-olds during a trachoma MDA in one health district. Monitoring data included the number of children reached and occurrences of adverse drug reactions. Feasibility, the extent to which the target population received the intervention (coverage), was assessed through a population-based, household survey with parents/caregivers of eligible children conducted after the MDA. Acceptability was explored through in-depth interviews (IDIs) with parents/caregivers of eligible children, focus group discussions (FGDs) with community drug distributors (CDDs), and IDIs with their supervisors. CDD FGDs and supervisor IDIs also documented implementation challenges and recommendations for scale-up. 1,735 1–5 month-olds received azithromycin during the pilot activity (estimated population coverage of 90.2%). Adverse drug reactions were reported for 1% (n = 18) infants; all were mild and self-limited. The post-MDA coverage survey interviewed 267 parents/caregivers; survey-based intervention coverage was 95.4% of 1–5 month-olds. Qualitative data revealed high intervention acceptability among parents, CDDs, and supervisors. Implementation challenges included the need to weigh babies to calculate dosage for 1–5 month-olds and the need to obtain written informed consent from parents to provide the drug to 1–5 month-olds. CDDs also indicated the need for more information on azithromycin and possible side effects during training. Delivering azithromycin to younger infants appears acceptable to parents and implementers; >90% coverage indicates feasibility to integrate into a trachoma MDA. (Clinicaltrials.gov ID number: NCT04617626).

## Introduction

Although considerable progress has been made to reduce under-5 and infant mortality rates globally, certain regions, West Africa in particular, continue to experience disproportionately high under-5 mortality rates. Even in countries in the West Africa region that have achieved under-5 mortality rates less than 80 per 1,000, some still have sub-national areas where mortality remains high [1]. For example, as of 2019, the national under-5 mortality rate in Côte d’Ivoire was estimated to be 79 deaths per 1,000 live births [1]; however, some regions within the country, particularly the north, experience, rates greater than 100 deaths per 1,000 live births [2].

Growing evidence suggests that bi-annual, mass administration of single-dose azithromycin (MDA-azithromycin) reduces child mortality among young children in high child-mortality settings. The *Macrolides Oraux pour Réduire les Décès avec un Oeil sur la Résistance* (MORDOR) (in English, oral macrolides to reduce deaths with an eye on resistance) trial examined the effect of bi-annual MDA-azithromycin on all-cause mortality among children aged 1–59 months in Malawi, Tanzania, and Niger [3–5]. The study found that child mortality was 13.5% lower in communities that received the intervention compared to control communities, with the greatest mortality reduction observed in Niger, a country with the highest overall baseline mortality [3–5]. Results also suggested that children aged 1–5 months benefitted the most with up to 25% reduction in mortality [3–5]. Based on MORDOR study findings, the World Health Organization (WHO) developed guidance to countries that outlines recommendations for the use of biannual, MDA-azithromycin targeting children from 1 through 11 months to reduce child mortality in settings where infant mortality rates exceed 60 deaths per 1,000 live births, or under 5 mortality exceeds 80 deaths per 1,000 live births [6]. In response to the WHO guidance on MDA-azithromycin, strategies to efficiently deliver the drug to young children are being explored.

One platform that has potential to be leveraged is that currently used for the prevention and treatment of *C*. *trachomatis* (trachoma) infection. Trachoma is the leading infectious cause of blindness worldwide and its areas of endemicity overlap with many regions that have high rates of under-5 child mortality including many of the poorest areas of Africa [7]. Since 1993, the WHO has led the campaign to eliminate trachoma through its SAFE strategy, which includes Surgery, Antibiotics to treat and prevent infection, Facial cleanliness, and Environmental improvement [7]. The antibiotic arm of the strategy involves annual delivery of a single-dose azithromycin to all community members ages 6 months and older in trachoma endemic areas [7]. Children under 6 months of age and pregnant women are offered tetracycline eye ointment in place of the azithromycin [7]. Given its design, the use of the same medication, and overlapping target population, trachoma mass drug administrations (MDAs) could be an optimal platform for delivering MDA-azithromycin to children.

Although the trachoma MDA platform holds many advantages for delivering MDA-azithromycin to children, there are several adaptations that would be required to the existing approach. Azithromycin distribution would need to be extended to include 1- to 5-month-olds who currently receive tetracycline ointment under the trachoma program [8]. The dose of azithromycin for children 6 months to 7 years for trachoma elimination is determined using height-based dosing guidelines, but no guidelines exist for height-based dosing of children under 6 months [8]. Those who administer the drug would need to be equipped and trained to use a weight-based dosing approach for younger infants. Additionally, because the trachoma MDA takes place once yearly, a second, stand-alone MDA-azithromycin would need to be implemented each year targeting only 1- to 11-month-old children. Successful integration of MDA-azithromycin into the trachoma MDA platform will require evidence to understand the most effective and efficient ways to implement required adaptations to the platform.

The aim of this pilot study was to examine the operational feasibility and acceptability of leveraging the existing trachoma MDA platform to expand delivery of single-dose azithromycin during a single round of an annual trachoma MDA campaign to children 1 to 5 months-old in one high child mortality health district in Côte d’Ivoire. As such, the pilot study was designed to: 1) examine the extent to which activities were carried out as planned (fidelity) during the pilot implementation; 2) estimate intervention coverage (reach) among the target population; 3) assess level of understanding and acceptability of the strategy among those involved in implementation and among the parents/primary caregivers of children targeted by the intervention; and 4) document implementation challenges to make recommendations for scale-up.

## Methods

### Ethics statement

The study was reviewed and approved by the National Research Ethics Committee of Côte d’Ivoire (study number 111-21/MSHP/CNESVS-km) and FHI 360’s Protection of Human Subjects Committee (study number 1575564). The study was registered on 05/11/2020 at clinicaltrials.gov, ID number NCT04617626. All methods were carried out in accordance with relevant guidelines and regulations. Consent was obtained for all participants. Written informed parental permission was obtained from all parents of children ages 1 to 5 months of age who received the azithromycin during the MDA. Written informed consent was obtained from all study participants who took part in IDIs or FGDs. Participants in the post-MDA coverage survey questionnaire provided verbal informed consent; waiver of written informed consent was obtained from the IRBs for the coverage survey due to the survey posing no more than minimal risk to the participant; no identifying data being collected from study participants and written documentation of informed consent being the sole link between the participant and the study data, incurring a potential risk of breach of confidentiality. Additional information regarding the ethical, cultural, and scientific considerations specific to inclusivity in global research is included in the Supporting Information (S1 Checklist).

### Study design

The pilot implementation study was carried out from November 2020 to February 2021 in one health district, located in north-western Côte d’Ivoire. Monitoring data on MDA implementation and adverse drug reactions were collected during the MDA that took place in November 2020. We conducted a population-based, post-MDA household survey with parents/primary caregivers of children aged 1 to 11 months in the pilot district in January 2021 to assess intervention coverage. The survey was followed by in-depth interviews (IDI) with a subset of participating parents/primary caregivers to examine intervention acceptability. Focus group discussions (FGD) with community drug distributors (CDDs) and IDIs with their supervisors explored implementer experiences including challenges to implementation and acceptability of expanding delivery of azithromycin to the youngest infants.

### Study setting

The study was conducted in Boundiali Health District in the Bagoué Region in north-western Côte d’Ivoire, which has a population of 156,695 (data provided by the Côte d’Ivoire National Institute of Statistics from 2019), including approximately 4,929 children under 11 months. The district is largely rural, and is comprised of 1,867 villages ranging in size from approximately 300 people to an estimated 40,000 in Boundiali proper. Ongoing interventions aimed at addressing under-five mortality include expanded immunization program for children 0 to 11 months, malaria prevention during pregnancy and use of insecticide treated bed nets for children, vitamin A supplementation and deworming, and implementation of essential family care practices. This health district was selected by the National Neglected Tropical Disease Program (NTDP) for the pilot study because it met the high mortality criteria, was implementing trachoma MDAs, was geographically accessible, and had a trachoma MDA scheduled during the study period.

### Intervention description

The NTDP is responsible for leading MDAs to treat trachoma and other neglected tropical diseases in endemic regions in Côte d’Ivoire. Responsibility for drug distribution during an MDA has been decentralized to health districts and is led by the District Health Director’s office. MDAs are carried out by teams of CDDs and their supervisors working in catchment areas for the health care facilities within districts. Typically, CDDs include community members who are engaged as community health workers and volunteers from the community. CDD supervisors are health care workers, usually nurses or midwives, employed by local health care services within the district.

Cascade training, from the national to regional to district to local levels, is used to prepare the MDA implementers. MDAs typically take place over the course of 5 days in a health district followed by a brief “mop-up” period to reach those missed if coverage targets are not achieved. CDDs, working in teams of three, deliver the drug to community members through either a door-to-door approach or by visiting areas in a town where people gather, such as a market or school. CDDs administer azithromycin in the form of a reconstituted liquid suspension dosed according to height/length for children ages 6 months to 7 years of age. Compensation for CDDs is determined by the NTDP and was not changed for this pilot study.

Adaptations to the trachoma MDA to integrate MDA-azithromycin were guided by a dedicated technical working group in Côte d’Ivoire led by the NTDP with members including representatives of various departments within the Cote d’Ivoire Ministry of Health and Public Hygiene, UN agencies, non-governmental organizations involved in the fight against NTDs in the country, research organizations, and training institutions. Principal changes to the trachoma MDA included: 1) adding weight-based dosing of azithromycin to infants from 1 month (>30 days) through 5 months of age; 2) altering MDA monitoring forms to capture azithromycin distribution to the 1–5-month-old age group; 3) implementation of informed parental permission to allow children ages 1 to 5 months to receive azithromycin in place of the tetracycline ointment; and 4) adding one additional CDD to each team of three CDDs to cover the additional work required. The ethics review committees determined that written informed parental permission was required because, although evidence exists as to the effectiveness of azithromycin to reduce child mortality, there is currently no approved label indication for this use. Although our study did not examine the effects of the drug, it was a study that included the off-label use of a drug as part of the intervention being examined, which required informed parental permission from parents before their child could receive the drug.

CDDs and all related supervisory staff were trained to administer weight-based doses of azithromycin at a dose of 20mg per kg of weight to children ages 1 to 5 months, rounded to the nearest 0.2ml. Handheld electronic scales equipped with removable, easy-to-clean baby slings were used to determine the child’s weight. A laminated dose card was used to identify the correct dose associated with the weight rounded to the nearest 0.5 kg. One additional CDD was added to each field team, resulting in a total of four CDDs per team. Teams typically cover about 100 individuals per day.

Following signed informed parental permission, the infant was weighed, and the appropriate dose of oral suspension was withdrawn from the container using a needleless, single-use syringe by a CDD. The drug was administered with the help of the mother, under a CDD’s direct observation. The azithromycin for the pilot intervention was the same as the oral suspension used during the trachoma MDA and was supplied in bottles containing azithromycin dehydrate powder equivalent to 1200 mg per bottle. The drug (trade name Zithromax) was donated by Pfizer Inc. (New York City, NY, USA).

### Sampling design, eligibility, and sample sizes

All children ages 1 to 5 months in the health district were recruited to participate in the MDA intervention. To be eligible for the pilot intervention, children had to reside in Boundiali Health District and be between 1 month (at least 30 days) to less than 6 months at the beginning of the week of the MDA campaign. Children below 3.0 kg (3rd centile for healthy 1-month old infants according to WHO growth charts) were excluded. Similarly, children who appeared severely ill at the time of the MDA, per the CDD’s assessment, had physical limitations for swallowing the drug delivered through the needle-less syringe, or had a known allergy to macrolide antibiotics were excluded.

Eligible participants for the coverage survey and parent/caregiver IDIs were mothers, fathers, or guardians, ages 18 years and older, of a child between the ages of 1 month (at least 30 days) and 11 months (i.e., not more than 365 days old) at the time of the MDA. Although azithromycin distribution for 6- to 11-month-olds is part of the trachoma MDA, we included parents of children in this age range because they will be targeted during the mid-year standalone MDA; we wanted to examine both coverage and acceptability for the full range of targeted children.

Eligible MDA supervisors included individuals who supervised CDDs in the pilot district during the MDA campaign and were aged 18 and older. Likewise, CDDs were eligible if they took part in the MDA in the pilot district and were 18 years and older.

For the coverage survey, a multi-stage cluster-sampling approach was used [9]. The primary sampling unit, villages, were selected randomly, probability proportionate to size, using the estimated population of the village as the size measure. Secondary sampling units were households with children ages 1–11 months. In each selected community, community health workers mapped all households with potentially eligible children and created a list of households for the study sampling frame, from which households were randomly selected without replacement. In households with more than one eligible child, one child was randomly selected as the index child for the survey. All eligible households (based on child age during the MDA) were included, regardless of whether the child had taken azithromycin.

Sample size for the coverage survey was based on an assumed estimated coverage of 50% with a 95% confidence interval of +/- 7.5% (the most conservative estimate). Without prior data to inform our estimate of intra-class correlation, we assumed a moderately conservative intraclass correlation coefficient of 0.05 to adjust the sample size for possible sampling design effects. Based on these assumptions, a total sample size of 250 (10 children per each of 25 sampled villages) was needed, resulting in a design effect of 1.45. The sample size was increased by 10% to account for possible non-response, resulting in a final sample size of 275, or 11 children per each of the 25 clusters/villages.

Participants for qualitative data collection were purposively selected from two rural and two urban areas among the 25 villages/areas covered by the post-MDA coverage survey. Parents who took part in IDIs were randomly selected with replacement from a list of parents who expressed interest in participating in the IDI during the coverage survey and provided contact information. Supervisors and CDDs were selected randomly with replacements from lists of those who took part in the MDA intervention. The number of qualitative interviews was limited to one FGD and three IDIs per area, giving an expected maximum sample size of 12 interviews with parents/caregivers, 12 with CDD supervisors, and 3 FGDs that included a total of 29 CDDs pulled from across the four areas.

### Data collection and measures

Monitoring data included a paper-based tracking log with the child’s unique study ID number, the child’s weight, dose of medication given, medication lot number, parent contact phone number (used by district focal persons to inquire about medication side effects), and any relevant observations, which was completed by CDDs each time an enrolled child 1–5 months was administered the dose of azithromycin. This monitoring data were used to calculate the recorded number of doses delivered. Government of Cote d’Ivoire population data were used to estimate the eligible population size. Aggregate summaries of age-specific adverse drug reactions (ADR) reporting during the MDA were also extracted for the study by district health teams from the ADR reporting forms completed in the pilot district.

The coverage survey took place from January 24–30, 2021, approximately 8 weeks after the MDA, and was delayed in part because of the December/early January holidays during time which many people travel. Following verbal informed consent, brief structured interviews were conducted by a team of two data collectors using a standardized questionnaire pre-programmed onto password-protected computer tablets. Survey topics included parents’/caregivers’ report of azithromycin uptake during the MDA, the child’s experience with any reaction to the medication within seven days after taking it, and for those children who did not receive the medication, reason why he/she did not. Other measures included: number of children under one year in the household and age; index child’s age and sex; respondent socio-demographic characteristics (sex, age, and education level); source of information on trachoma MDA prior to the event; whether or not the caregiver received any information on the use of single-dose azithromycin to reduce child mortality, and source of that information. Eligible households for which a primary caregiver was not present were revisited up to three times during the week of data collection before being counted as non-responsive.

IDIs and FGDs were conducted by a team of two trained research assistants to explore the acceptability of using the MDA platform for delivering azithromycin to younger infants, the degree to which MDA activities were carried out as planned during the pilot implementation, the factors affecting the processes and results of the MDA, and recommendations to improve the intervention strategy. Parent IDIs were conducted in French or in the local language. IDIs and FGDs audio recordings were transcribed and portions in local languages were translated into French.

### Adverse event monitoring

Adverse drug reactions (ADRs) were defined as undesirable effects possibly related to the drug that occurred within seven days of administration. Standard definitions of AEs and serious AEs were used to classify any reported events. Monitoring of ADRs and AEs was done through four mechanisms. During the MDA, the NTDP actively collected information on all ADRs, and AEs reported by community members who received the medication to CDDs, CDD supervisors, or the nearest health facility. Additionally, all the parents/caregivers of children 1–5 months who received azithromycin were called within one week after the MDA by focal persons within each village to inquire if the child experienced any side effects from the medication. If an ADR occurred, the focal person informed the nurse supervisors in their catchment area, who in turn informed the study investigators. Study staff also contacted all health facilities in the district weekly for four weeks (28 days) after the MDA to inquire if any children ages 1 to 5 months had sought treatment for potentially related symptoms. Lastly, during the coverage survey, participants were asked if their child experienced any ADR or other AE after taking the medication, if they required health care for drug side-effects, and if they sought and received care.

### Data analysis

MDA population coverage was estimated using monitoring data by calculating the proportion of children who received doses out of the estimated eligible population. Data from the post-MDA survey were descriptively summarized. Proportions and 95% confidence intervals (CI), adjusting for sampling design effect and sampling weights, were calculated for all survey data, except caregiver’s age (the only continuous variable), which was reported descriptively using the mean and 95% CI. All statistical analyses were conducted in SAS Enterprise version 8.3, including the calculation of sample weights and adjustments for sampling design using Proc survey methods [10].

For children who were subject of the post-MDA survey, we only collected month and year of birth. To calculate age group for these children, we assigned the 15^th^ (middle of the month) to all children and combined with their recorded month and year of birth to create an approximate date of birth. This date of birth was subtracted from the last day of the MDA to estimate the child’s age at the time of the MDA. Children were then grouped into a 1-to-5-month age group and a 6-to-11-month age group based on the calculated age at the time of the MDA.

French transcripts of qualitative data were translated into English; and analysis was conducted in both French and English. Transcripts were read by study investigators to clarify unclear data and identify recurrent themes. A priori codes related to the study objectives were applied to the data and additional deductive codes were developed based on emerging themes. An Excel data reduction matrix was used to organize the qualitative data and prepare the data for analysis. Once all the transcripts were coded, textual coding reports, including relevant quotes, were produced, and reduction techniques were used to examine codes in detail for sub-themes and patterns across the IDIs/FGDs. Finally, summary reports were developed including results across participant groups.

## Results

### Pilot MDA intervention

Overall, 1,735 children 1 to 5 months received azithromycin during the MDA, corresponding to a population coverage estimate of 90.2% according to monitoring data, based on the 2021 population estimates of eligible children provided by the NTDP just prior to the MDA campaign (Table 1). In comparison, the population coverage estimate for 6–11 months was 91.5%.

**Table 1:** Results of MDA event coverage, based on monitoring data.

Characteristic	Number (%)
Estimated number of eligible children ages 1–5 months^1^	1,923
Children 1 to 5 months who received azithromycin	1,735 (90.2%)
Estimated number of eligible children ages 6–11 months^1^	3,166
Children 6 to 11 months who received azithromycin	2,897 (91.5%)
1. Estimates for the numbers of eligible children are based on government census estimates.	

During the MDA campaign, 1% of children 1–5 months experienced any AE (diarrhea/loose stools, vomiting, fever) after taking the medication; these were reported to a CDD, an MDA supervisor or a health care worker (Table 2). No additional ADRs were reported through post-MDA monitoring calls to health facilities in the four weeks that followed the MDA. All recorded AEs during the MDA were identified in this manner and could be classified as non-serious, expected, and likely related to the azithromycin.

**Table 2:** Summary of AE reported during MDA event, by symptom reported (n = 1,735 children ages 1 to 5 months).

	% (n) (n = 1,735)
Experienced any adverse event or drug reaction	1.0% (18)
Experienced a severe adverse event	0% (0)
Reactions to medication reported^1^	
None	99.0% (1,717)
Cough	0.1% (2)
Runny/loose stools/diarrhea	0.3% (5)
Vomiting	0.3% (5)
Fever	0.1% (2)
Fever and diarrhea/loose stools	0.2% (3)
Vomiting and diarrhea/loose stools	0.1% (2)
1. % Does not total 100, more than one response was possible.	

### Post-MDA coverage survey

Of the 275 households selected for the survey, 267 parents or primary caregivers of children ages 1 to 11 months were interviewed across the sampled villages, representing 97% of the target sample size. Eligible individuals were not available to be interviewed in 5 sampled households, and two sampled households were deemed to be ineligible after sampling.

The sample of children was slightly greater for those 1–5 months-old compared to 6- to 11-month-olds (age at the time of MDA event in November 2020.) Most respondents (parent/caregiver) (84.3%) were female and had no formal education (75.7%; Table 3).

**Table 3:** Coverage survey participant characteristics (n = 267), non-adjusted, non-weighted.

Respondent Characteristic	% or mean (n = 267)
Mean Age (years)	30.2
Respondent’s sex	
Female	84.3% (225)
Male	15.7% (42)
Education (highest level completed)	
None	75.7% (202)
Any primary	13.1% (35)
Any secondary	10.5% (28)
Any post-secondary	0.8% (2)
Child’s age^1^	
1 to 5 months	57.7 (154)
6 to 11 months	42.3% (113)
Respondent’s relationship to index child	
Parent	88.4% (236)
Grandparent	6.7% (18)
Aunt/uncle	4.1% (11)
Co-wife	0.8% (2)

Survey-reported coverage with single-dose azithromycin was 95.9% (Table 4). Of the 11 infants who did not receive any medication during the MDA, most (n = 7) were not home at the time the team came by the house. In two cases, the infant was sick and could not receive the medication. Only in one case did the parent refuse because they were afraid of side effects. The remaining infant who did not receive medicine was because the parent was confused about needing the child’s health card to receive the medicine.

**Table 4:** Survey-reported MDA coverage and reasons for non-participation (n = 267).

Characteristic	%	95% CI
Azithromycin coverage, overall (ages 1 to 11 months)^1^	95.9	[92.9, 98.8]
Azithromycin coverage, ages 1 to 5 months	94.8	[90.5, 99.1]
Azithromycin coverage, ages 6 to 11 months	97.3	[94.3, 100.0]
Reasons child did not receive azithromycin^2^	(n)	
Child not home during MDA	7	
Child was sick during MDA	2	
Parent refused due to concerns about side effects	1	
Parent thought health card was required to receive drug	1	
1. Result accounts for sampling weights and clustering by village. 2. Counts only presented for reasons for not receiving azithromycin		

According to parent/guardian reports, most children were dosed according to MDA and study protocols (Table 5). In a few instances, children who were 6 months or older at the time of the MDA were dosed using a weight-based dose, which, while not standard MDA practice, is an acceptable dosing approach, so were categorized as correctly dosed. Seven percent of parents reported that their children under 6 months had doses calculated using height/length measurement, which would not be correct for this group. A minority of respondents reported that the child was both weighed and measured and a few also reported that the child was neither weighed nor measured to calculate the dose. In one case, the parent reported that the child’s weight was taken from a recent reading recorded in their health card.

**Table 5:** Survey-reported azithromycin dose calculation, by age group among those who received azithromycin during the MDA (n = 256), weighted and adjusted for sampling design effects.

Characteristic	1–5 months old % [95% CI]^1^	6–11 months old % [95% CI]^2^
Dose was calculated using appropriate method	84.9 [74.8, 94.9]	91.7 [82.5, 100]
Way dose was calculated		
Weigh the child	84.1 [74.1, 94.2]	23.2 [11.2, 35.2]
Measure the child’s height/length	10.4 [2.8, 17.9]	66.3 [52.4,80.2]
Both weighed and measured the child	0.7 [0.0, 2.3]	2.1 [0.0, 5.3]
Neither weighed nor measured the child	3.4 [0.0, 9.1]	7.5 [0.0, 16.0]
Don’t know	1.4 [0.0, 3.3]	0.9 [0.0, 2.7]
1. 8 missing 2. 3 missing		

Very few adverse drug reactions (ADRs) were reported in the coverage survey (Table 6). The majority of ADRs identified during the survey were considered mild by the parents and not reported to any health authority. Among those who did not report the reaction, the majority (18 of 20) said it was because the side effect was too minor. Among the 10 respondents who did report their child’s reaction, one said the symptoms resolved on their own, 5 sought medical care, and 4 treated the symptoms with traditional medicines. Of the 5 who sought medical care for the reaction, 4 received medicine at the health facility and 1 was examined by a health care worker but received no treatment.

**Table 6:** Survey-reported results of adverse drug reactions (parent report) among those who received azithromycin (n = 256), weighted and adjusted for sampling design effects.

Characteristic	%	95% CI
Experienced any adverse drug reaction (% and 95% CI)	11.6	[5.7, 17.4]
Experienced a serious adverse drug reaction (% and 95% CI)	0	-
Reported ADR	3.8	[1.6, 6.1]
Reactions to medication		
None	88.4	[82.6, 94.3]
Runny/loose stools/diarrhea	6.2	[0.9, 11.5]
Fever	3.1	[1.1, 5.5]
Vomiting and diarrhea	1.1	[0, 2.9]
Vomiting	0.8	[0, 1.9]
Fever and diarrhea	0.4	[0, 1.0]
Symptom resolution among those experiencing an ADR	*(n = 30)*	
Resolved on own	70.0	
Sought medical care	16.7	
Used traditional medicine / sought traditional healer’s help	13.3	
All results account for sampling weights and clustering by village.		

### IDIs and FGDs

#### Acceptability of administration of azithromycin to babies 1–11 months old

Providing azithromycin to 1- to 11-month-olds was highly acceptable to parents, CDDs and supervisors with participants noting both that they were comfortable with children receiving the medication and that children would benefit from azithromycin. Additionally, some parents said that improving child survival benefits parents and the larger community with one parent noting: *"If the child has a long life*, *if they become someone tomorrow*…*we all benefit*.*"* Several supervisors noted that azithromycin was beneficial and addressed other health concerns in addition to trachoma. Similarly, supervisors described azithromycin favorably using terms such as it being *“beneficial”* or “*good for kids”* to describe it.

No parents expressed concerns about their child taking the medication. The main concern, expressed by both CDDs and supervisors, focused on the potential for side effects. Several supervisors noted that prior to the MDA they were concerned about side effects and to best manage them if they occurred, especially for infants between one and five months of age. One supervisor said they wanted *"to know if it was going to have a lot of side effects… since we were putting it in children one to five months old*.*”* After the MDA, however, both supervisors and CDDs noted that the experience alleviated their concerns regarding safety and side effects.

*"For me personally as a health officer, I think it was good. At first, I was a little apprehensive. During the training, we were told that the administration of azithromycin in children under 6 months of age in many countries showed that this could have significantly reduced the mortality rate… . And after the campaign, since no household has reported any signs of side effects, I think that’s a good thing. I’m really satisfied."*

*- Supervisor*

*“Well, me personally, I didn’t have any concerns… Because it’s true that it was my second time to participate in a campaign like that… I knew the importance, now to reassure even my parents… There was confidence in this medication. "*

*- CDD*

CDDs also described general concern over azithromycin initially because it was a new medicine for babies, but their concerns were overcome after the MDA when they saw that it was safe. CDDs reported encountering several parents who were hesitant for their children to take the medication or who refused due to: concerns about side effects, particularly diarrhea and fever; concerns about the cost of treating side effects, or the overall safety of the medication. In contrast, no parents expressed concerns during IDIs and several noted that receiving the medication as part of an MDA or through a local health facility eliminated any concerns. One parent noted “*If the medication arrives*, *we do not worry*, *we do not hesitate to go take it;”* and most said they had no unanswered questions about the medication. It should be noted that all parents interviewed had accepted the medication for their children and that the parental refusal rate was very low. A few supervisors mentioned they encountered a few parents who were suspicious of the MDA in the context of COVID-19.

*"Yes, yes, we had other difficulties to manage because we are also in a context of COVID-19 so for that, with everything that was said on social networks, the parents were still a little skeptical. So, when we went to meet them, they already had prejudices. There was also a difficulty there."*

*- Supervisor*

#### MDA implementation

Overall, many supervisors expressed positive feelings about the MDA, some noting that it *‘went well*,*’* was *‘successful*,*’* or needed to be extended to other parts of the country. The most common concerns reported were per diems for CDDs, the process of weighing and dosing infants, administering informed consent and pre-MDA message dissemination. All CDDs and most supervisors described the current per diems as being insufficient. One supervisor noted how it reflected the level of respect with which CDDs were regarded.

“*It’s when you call someone to give 2000F [3*.*63 USD]*, *it’s not easy*, *eh*. *Often*, *it’s shame*. *How they respect us and there you go*! *"*

*- Supervisor*

Another supervisor identified the low compensation as a reason why it was challenging to recruit CDDs with greater education or qualifications. In addition to increases to per diems, both CDDs and supervisors advocated for the provision of phone airtime and travel expenses to CDDs to pay for fuel while travelling for MDA campaigns.

Supervisors and CDDs both felt that the scales used to weigh infants were inaccurate and difficult to use. They reported that they did not feel comfortable weighing babies in them either because they saw the scales as poorly made or because they were uncomfortable with lifting the babies in the sling. Some mentioned needing to weigh infants multiple times to obtain accurate weights. Some supervisors felt that weighing children was a new activity that added to the CDDs’ workload. One CDD noted:

*"What you did that wasn’t good for me was the scales…The scales were small. It was not good. It could break since the iron was not very strong. We ourselves were afraid to measure the child."*

*- CDD*

Calculating and preparing dosages of azithromycin was also raised as an issue by some supervisors with a few mentioning that calculating dosages was difficult for some CDDs and others citing confusion over the amount of water needed to dilute the azithromycin powder or concerns with obtaining accurate weight required for dosing.

Supervisors reported some difficulty with completion of the informed consent forms, because of the length of time it took to administer due to the low literacy level of parents. Some supervisors also stated that parents found the form difficult to understand and questioned why they must sign the form, given that parents did not need to sign permission for children ages 6 months and older to receive the same medication. As described by one supervisor:

*"Well, compared to informed consent, about the parents of the children, it was really a bit complicated. They couldn’t understand why they had to sign. So, if we could improve that process a little bit."*

*- Supervisor*

Additionally, both CDDs and supervisors reported issues with completing the data collection forms in general including the number of required forms and keeping forms organized.

#### Suggestions for improvement

**Fig 1:**
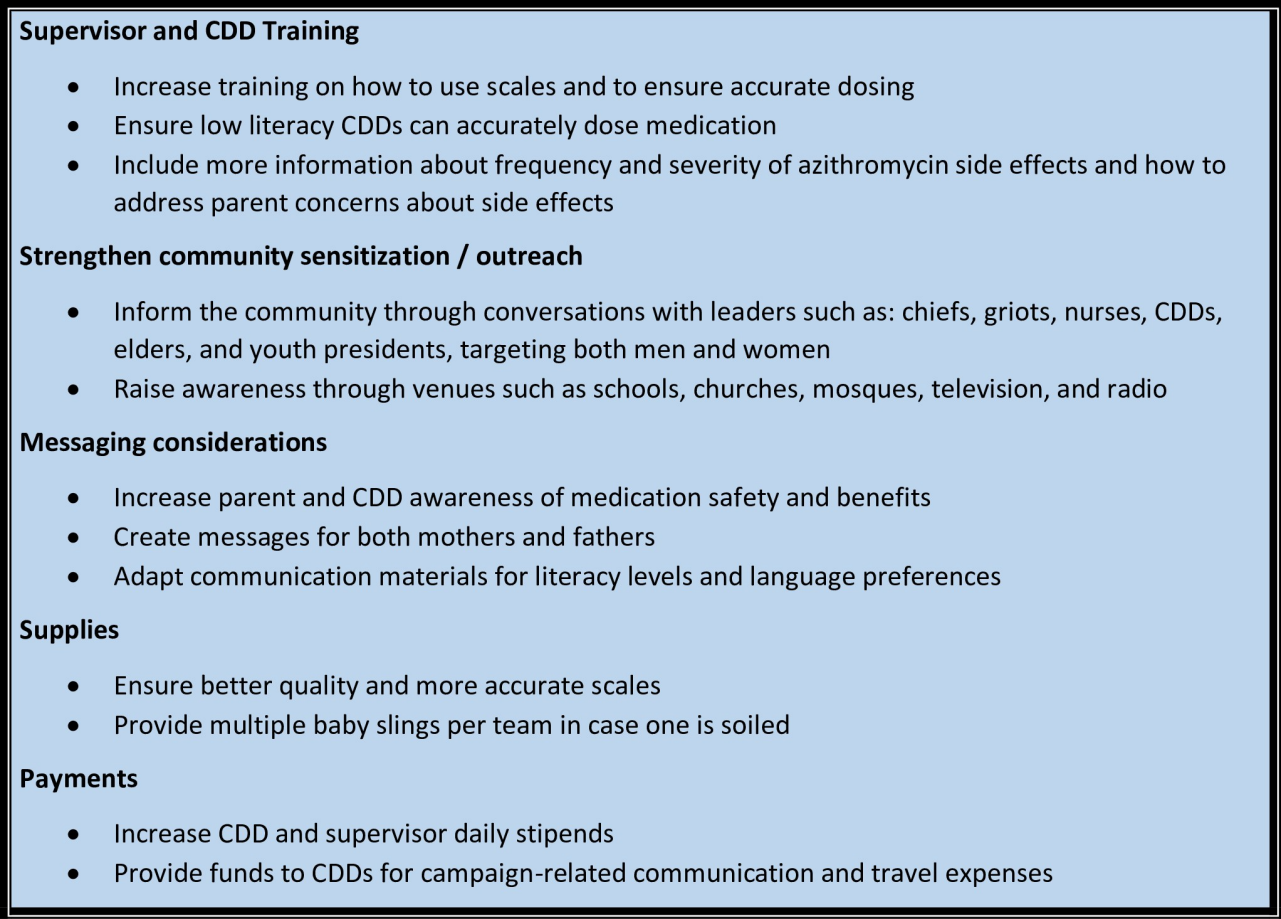
Key Suggestions for azithromycin administration during MDAs.

Supervisor and CDD participants suggested ways to improve the MDA, focusing on training, outreach and sensitization, messaging, supplies, and payments. These are outlined in Fig 1.

## Discussion

This study was the first, of which the authors are aware, to examine the feasibility and acceptability of using an established MDA campaign platform to reach infants with single dose azithromycin for the purposes of reducing child mortality in high mortality settings. Results from this pilot study demonstrate that extending mass administration of azithromycin to infants ages 1 to 5 months appears to be feasible and can be successfully integrated into an existing trachoma MDA platform. Coverage of the targeted population of 1- to 5-month-olds exceeded 90% by both monitoring data and post-MDA coverage survey estimates. Even though parents of this age group were offered the standard trachoma treatment (tetracycline ointment) as an option, and choice of the azithromycin required signed informed parental permission, nearly all parents accepted azithromycin for their eligible children, based on both survey results and monitoring data. Additionally, CDDs were able to not only administer the required informed consent, but also overwhelmingly used the correct procedures for calculating the child’s dose of azithromycin, based on survey results. It is important to note that these results were obtained in a setting where the number of CDDs was increased by 33% by the study–one additional CDD added to each of the existing 299 teams of 3 CDDs for the trachoma program. When taken to scale in multiple districts, where the numbers of CDD teams can be in the thousands, it may not be financially feasible to maintain the proportionate increase of CDDs, particularly when the target population– 1- to 5-month-old children–makes up roughly only about 1.5% if the population being targeted for the trachoma MDA.

We also found that MDA azithromycin for child survival was highly acceptable for all key stakeholders. Parents, CDDs, all reported positive perceptions for the use of azithromycin among 1 to 5 month-old children to reduce child mortality. Even some CDDs who reported being initially hesitant to provide the drug to the younger age group, stated their concerns were alleviated over the course of the MDA.

Despite high acceptability and overall feasibility demonstrated in this pilot study, a number of challenges were documented that will need to be addressed or could hinder larger scale implementation. Fidelity to implementation is a key consideration for scalability and one key operational challenge to distributing the drug was identified by several CDDs who voiced concerns over having to weigh younger infants to calculate the appropriate dosage for the medication. In addition to adding a step in the process of delivering the medication, weight-based dosing also required the project to procure one portable scale per CDD team, which adds to the cost of the MDA. According to survey data, most children in both the 1-to-5-month and 6-to-11-month age groups were dosed using an appropriate technique; however, approximately 13% of children in the younger age group had their height/length measured rather than being weighed, which could lead to over- or under-dosing of the medication, the effects of which are not known. Weight-based dose calculation, while a departure from standard MDA procedure, does permit more accurate dosing when done correctly, and the current height-based dosing guidelines do not include children under 6 months of age [11, 12]. A recent article from members of the same team who led the earlier MORDOR trials suggests an age-based dosing approach could be used for children under 6-months of age. This new dosing approach used dose tolerance ranges of 10mg to 20mg of azithromycin per kg of weight for children ages 1 to 2 months, and 15 mg to 30 mg of azithromycin per kg of weight [13]. While this age-based dosing approach may hold promise, all of the clinical trials on which the current WHO guidance for azithromycin to reduce child mortality are based on a dose of 20 mg of azithromycin per kg of weight [6]. More research is needed to understand how these wider dose tolerance ranges impact the mortality outcome associated with the intervention, as well as potential effects on antimicrobial resistance to the drug. Alternate strategies, such as developing height-based dosing guidelines for the younger infants or identifying other ways to efficiently weigh children while conducting community distribution of a drug, should also be explored.

The safety of any medical intervention is always an essential consideration, and an even greater concern for vulnerable populations such as infants. Although there is no current label indication for the use of azithromycin in children under 6 months of age, it is used off-label for a variety of reasons in children in this age group [14, 15]. Azithromycin is usually well-tolerated; typical drug reactions in pediatric patients are mild and self-limited, with most common side effects experienced in clinical trial data being diarrhea or loose stools (4.3%), abdominal pain (1.4%), nausea (1.0%), vomiting (4.9%), and rash (1.0%) [16, 17]. Our findings, consistent with those from the MORDOR and related trials, indicate a low number of mild and self-limited adverse drug reactions in the target population [4]. Additional studies have found that the drug appears to be safe when used among younger infants (< 6 months) [18–20]; however, there is some evidence suggesting that infantile hypertrophic pyloric stenosis may be a concern when the drug is used among very young infants (neonates) [19]. In response to this concern, the WHO has recommended that the intervention target children ages 1 months and older. Scale up of this intervention will require continued monitoring of adverse drug reactions and serious adverse reactions to ensure the benefits of the intervention continue to outweigh risks; however, our pilot experience demonstrated that the additional ADR monitoring activities implemented (calls to parents of 1- to 5-month-olds and calling health facilities weekly for four weeks) did not yield any additional cases of ADRs, thus routine monitoring conducted during the MDA appears to suffice for future efforts.

Obtaining informed parental permission prior to administering the drug for children under six months was seen as a logistic burden by CDDs; however, given the lack of a label indication for use of the drug among children under six months of age, offering the drug in the context of research and obtaining informed parental permission are necessary. Moving forward, it will be important to continue to improve the consent process, ensuring all critical information is conveyed in sufficient detail, while in a manner that is readily understood by parents and easy to administer by drug distributors. Additionally, it would be ideal to identify policy options that would officially support the use of azithromycin for this purpose in children under 6 months of age in a non-research context.

One matter beyond the scope of this pilot study, but of concern for future scale-up, is the impact of MDAs on antimicrobial resistance (AMR) [21–36]. There is serious concern about an expanded threat of AMR associated with MDAs [21–36]. Because of its cost, azithromycin is not widely used in many of the high infant/child mortality settings that would be targeted by MDA-azithromycin, but it remains an important tool in the antibiotic arsenal for several severe infections, such as community acquired pneumonia [37]. Research to date has demonstrated at least a transient increase in AMR to azithromycin in communities where trachoma elimination programs have taken place or where the original clinical trials of the MORDOR research was conducted [21–36]. Careful monitoring of trends in AMR related to azithromycin use will be essential to understanding both the long-term and short-term impacts of the intervention on AMR.

Lastly, at the time the study was conducted, the trachoma program in Côte d’Ivoire overlapped with nearly half of areas where under-5 mortality met the threshold for the MDA azithromycin intervention, based on WHO guidance. In Côte d’Ivoire, 14 regions, including 46 health districts, met the under-5 mortality threshold, a total of 19 of which were endemic for trachoma. This overlap allowed the study to leverage an existing activity for distribution of azithromycin to the targeted age group of 1 to 11 months. However, the trachoma MDAs were only scheduled annually, which necessitates a strategy to reach the same population a second time in a year in the absence of the trachoma MDA. Additionally, the trachoma platform did not reach more than half of the districts with high under-5 mortality. These facts, coupled with progress toward trachoma elimination, which will end the need for trachoma MDAs, require identification of alternate effective and cost-efficient strategies to deliver MDA-azithromycin to the largest proportion of eligible children.

### Limitations

While results from this study provide important insight into factors that will need to be considered as MDA-azithromycin is rolled out and scaled up across different settings, it is not without some limitations. First, findings from this study are limited to the context of an integrated trachoma MDA, and further to the one specific setting within Côte d’Ivoire; however, many of the lessons learned may be applicable to similar mass campaigns into which MDA azithromycin might be integrated. Also, our primary feasibility outcome measure–the extent to which the target population received the medication–relied on two different sources, both of which have inherent limitations. Calculating coverage using doses distributed as the numerator and population estimates as the denominator, can lead to over-estimated coverage, particularly at local levels [38]. The second method, a population-based coverage survey, used a rigorous approach to obtain a probability sample to estimate coverage; however, population-based coverage surveys also have limitations, such as relying on population estimates for the selection of primary sampling units [38]. Both methods revealed consistently high coverage, but results must be interpreted in the context of the limitations of each method. Lastly, our outcome measure in the coverage survey relied on parent/guardian reports of whether the child received the medication during the previous MDA. Self-report of an outcome can be subject to certain biases, such as social-desirability bias, and the extent of the influence of such bias is not known. Results from our qualitative inquiries, however, revealed very high acceptability among parents, providing further support for our survey findings on coverage.

## Conclusion

This pilot study found MDA of azithromycin to younger infants could be effectively integrated into a trachoma MDA platform when limited to one health district. With an estimated coverage of greater than 90% of the target audience, the pilot activity was successfully implemented; however, the intervention was not without a number of important challenges that will need to be addressed as the intervention is brought to scale. This study provides valuable insight into the factors necessary to successful implementation within the context of an existing trachoma MDA platform, including identifying of potential challenges to taking the intervention to scale, as well as providing guidance on how to address these issues.

## Supporting information

S1 Checklist Inclusivity in global research. (DOCX)
